# The President's Address

**Published:** 1900-08-04

**Authors:** William Alfred Elliston

**Affiliations:** Senior Surgeon East Suffolk and Ipswich Hospital.


### British Medical Association.

### THE PRESIDENT'S ADDRESS.

By William Alfred Elliston, M.D., Senior Surgeon East Suffolk and Ipswich Hospital.

In welcoming the members of the British Medical Association assembled at Ipswich, Dr. Elliston said it was 26 years since they had met in East Anglia—that time in the city of Norwich—and it spoke eloquently for the ever-growing importance and utility of the Association that while on that occasion it possessed only about 6,000 members, and the scientific business of the meeting was conducted in four sections, on this occasion the membership had increased to over 18,000, of whom 4,000 were members of our Colonial branches, and the scientific work to be transacted needed 13 sections for its due consideration.

After mentioning shortly some of the attractions of Ipswich, and paying a tribute to the memory of those who had passed away since the time of the last meeting, Dr. Elliston proceeded to give a general review of the development of British medicine and the evolution of the modern physician.

The continuous progress of British science commenced, he said, with the return of Thomas Linacre to Oxford from the Italian universities, whence he came imbued with what was termed the "new learning," then recently introduced by the Greek professors, who had been attracted to Italy. At this time the learned of all countries were profoundly impressed by the recent discovery of America, by the scientific discoveries of such men as Copernicus and Paracelsus. The printing press was changing the conditions of life, and literature became the common property of all.

The great event in the sixteenth century, so far as medicine is concerned, was the success of Thomas Linacre in persuading the King to grant a charter to a small body of medical graduates, who were thenceforth called the Royal College of Physicians. The charter of the Royal College of Physicians was granted September 23rd, 1518. In 1540 the Barbers and Surgeons were united by Act of Parliament 38 Henry VIII., cap. 42. They were incorporated as the "Maisters or Governors of the Mysterie and Commonalitie of Barbers and Surgeons of London." In 1543 the irregular practitioners of London were protected by Act, and became one of the corporations of that city, and both by Royal charters and by Acts of Parliament exercised the power of granting licence to practice medicine. In 1505 the Royal College of Surgeons of Edinburgh was founded, and in 1599 the Faculty of Physicians and Surgeons of Glasgow.

Thus, in the sixteenth century there were already established physicians, surgeons, and apothecaries. The surgeons were associated in their guild with the barbers, and the apothecaries with the grocers. While the physicians were men of education, cultured according to the education of the time, the surgeons and apothecaries were not a very highly educated class, and they remained so, with few exceptions, until the rise of the nineteenth century.

It was at the very commencement of the seventeenth century that the original research of two great English physicians, Gilbert and Harvey, completely altered the position of our country in scientific advance.

The results of Harvey's observations are the more remarkable when we consider the scanty instruments and appliances then available, contrasted with our own time, when the senses, the sciences, and the arts are all brought into requisition for the discovery and analysis of objective symptoms.

The microscope was as yet undiscovered. The number of pulse beats was not measured by a watch until nearly a century after Harvey's time. In his day, according to Dr. Norman Moore, physicians contented themselves with estimating the character of the pulse rather than its precise rate. Specialism as now understood did not exist, and Harvey was a surgeon as well as a physician. It was about this time that a small body of students assembled at Oxford in 1645, who were later, in 1662, to be known as the Royal Society.

During the seventeenth century practical medicine in Great Britain was gradually developing; a pharmacopœia had been issued; cinchona bark was introduced; botanical gardens were established. The teachings of Francis Lord Bacon, and subsequently John Locke, influenced the direction of thought, while the great learning and superb common sense of that great physician, Sydenham, gave effect to the general advance of this period.

In surgery the growth was slow, principally through the general want of knowledge of anatomy acquired by dissection; but two Englishmen had distinguished themselves in this branch—Nathaniel Highmore and Thomas Wharton.

At the beginning of the eighteenth century a warm contest arose between the physicians and apothecaries, the former accusing the apothecaries of usurping their province, the latter continuing and justifying the usurpation, until the matter was formally settled by the House of Lords in 1703, when it was decided that the duty of the apothecary not only consisted in compounding and dispensing, but also in directing and ordering the remedies employed in the treatment of disease.

Medical education, however, lagged sadly in England for a long time. At the beginning of the eighteenth century there was practically no systematic medical training in the British Isles. Isolated courses of lectures were given in connection with some of the hospitals, but it was not until 1766 that Rutherford was appointed Lecturer in Medicine in Edinburgh, where until 1785 he delivered his lectures in Latin. He founded clinical teaching in Edinburgh, and his success was so great that the manager of the Royal Infirmary appointed a special ward for his exclusive use, thus laying the foundation of that form of teaching in which Edinburgh has since held proud pre-eminence. The scantiness of the means of medical education which were available in London during the eighteenth century is remarkable. Until the last quarter of the century there was no means or possibility of obtaining a complete medical education in London. Surgeons and apothecaries were supposed to be able to pick up a sufficient smattering of their work by attending a hospital for a few months, and physicians were educated elsewhere. Some half a dozen took a degree at Oxford or Cambridge, and these alone could become Fellows of the Royal College of Physicians; others, a much larger number, went to Edinburgh and the Scotch Universities, Dublin, or abroad.

It was not till 1745 that the Barber Surgeons were dissolved, and were incorporated as distinct societies, 18 George II., c. 15. To us it is inconceivable that this union could have lasted so long. The barber's shop was a favourite resort for idle persons, and, in addition to its attraction as a focus of news, a lute, viol, or some such musical instrument was usually kept for the entertainment of waiting customers.

The surgeons of the eighteenth century, like the physicians, were accustomed to give advice at taverns. Sir William Blizard, who was President of the Corporation of Surgeons in 1787, was one of the last survivors in this line of practice. For many years he is stated to have attended regularly at Batson's Coffee House in Cornhill at a certain time in the day to hold consultations.

Coming to more modern times and the more recent developments of medicine and surgery, Dr. Elliston, speaking of the early history of ovariotomy, said: "It is not, perhaps, so generally known as it deserves to be that William Jeaffreson, of Framlingham, in the county of Suffolk, an East Anglian surgeon, was the first surgeon in England to perform the operation of ovariotomy by a small central incision. His friend and neighbour King at Saxmundham, Suffolk, about ten miles away, operated a few weeks later, with complete success, his incision being somewhat longer—about three inches. Two years subsequently a third East Anglian surgeon, Crisp, of Harleston, who lived about twenty miles from Jeaffreson, was also successful, with an incision of two inches. These three cases, which were performed by three surgeons in general practice in this county, were the first in England, in response to the doctrines of William and John Hunter, and are the more remarkable when we consider that no operation of the kind was attempted in London until four years later, and no ovariotomy was performed in a London hospital until ten years after Jeaffreson's case."

Speaking of the introduction of anæsthetics, he referred to the account given by Mr. William Cadge of the first administration of ether in England; and, finally, after tracing the evolution of the modern physician, Dr. Elliston referred to the expense of a modern medical education. Medicine, so far as education is concerned, is, he said, certainly the most costly of the learned professions, this being due in large degree to the long period of time required for the medical curriculum, and to the multiplication of qualifications which he regarded as a fashionable absurdity.

